# A Perspective on Plant Phenomics: Coupling Deep Learning and Near-Infrared Spectroscopy

**DOI:** 10.3389/fpls.2022.836488

**Published:** 2022-05-20

**Authors:** François Vasseur, Denis Cornet, Grégory Beurier, Julie Messier, Lauriane Rouan, Justine Bresson, Martin Ecarnot, Mark Stahl, Simon Heumos, Marianne Gérard, Hans Reijnen, Pascal Tillard, Benoît Lacombe, Amélie Emanuel, Justine Floret, Aurélien Estarague, Stefania Przybylska, Kevin Sartori, Lauren M. Gillespie, Etienne Baron, Elena Kazakou, Denis Vile, Cyrille Violle

**Affiliations:** ^1^CEFE, Univ Montpellier, CNRS, EPHE, IRD, Montpellier, France; ^2^CIRAD, UMR AGAP Institut, Montpellier, France; ^3^UMR AGAP Institut, Univ Montpellier, CIRAD, INRAE, Institut Agro, Montpellier, France; ^4^Department of Biology, University of Waterloo, Waterloo, ON, Canada; ^5^Center for Plant Molecular Biology (ZMBP), University of Tübingen, Tübingen, Germany; ^6^Quantitative Biology Center (QBiC), University of Tübingen, Quantitative Biology Center (QBiC), University of Tübingen, Germany; ^7^Biomedical Data Science, Department of Computer Science, University of Tübingen, Tübingen, Germany; ^8^BPMP, Univ Montpellier, CNRS, INRAE, Montpellier, France; ^9^CEFE, Univ Montpellier, CNRS, EPHE, Institut Agro, IRD, Montpellier, France; ^10^LEPSE, Univ Montpellier, INRAE, Institut Agro, Montpellier, France

**Keywords:** *Arabidopsis thaliana*, near-infrared spectroscopy (NIRS), multivariate analysis, machine learning, functional traits, metabolomics, trait-based ecology

## Abstract

The trait-based approach in plant ecology aims at understanding and classifying the diversity of ecological strategies by comparing plant morphology and physiology across organisms. The major drawback of the approach is that the time and financial cost of measuring the traits on many individuals and environments can be prohibitive. We show that combining near-infrared spectroscopy (NIRS) with deep learning resolves this limitation by quickly, non-destructively, and accurately measuring a suite of traits, including plant morphology, chemistry, and metabolism. Such an approach also allows to position plants within the well-known CSR triangle that depicts the diversity of plant ecological strategies. The processing of NIRS through deep learning identifies the effect of growth conditions on trait values, an issue that plagues traditional statistical approaches. Together, the coupling of NIRS and deep learning is a promising high-throughput approach to capture a range of ecological information on plant diversity and functioning and can accelerate the creation of extensive trait databases.

## Introduction

In trait-based ecology, the comparison of plant phenotype across multiple species aims at identifying general trends of variation to describe the biodiversity of plant forms and functions (Grime, 1988; Keddy, 1992; Díaz et al., 2016; Garnier et al., 2016). Ecological strategies are characterized qualitatively and quantitatively from the measurement of key functional traits, i.e., morphological, physiological, and phenological parameters that determine plant growth and reproduction (Violle et al., 2007). However, our understanding of plant diversity with comparative approaches is impeded by three main limitations. First, measuring the traits that describe ecological strategies on many individuals remains laborious. Second, intraspecific trait variability and plasticity to the environment still remain largely unconnected to traditional cross-species studies (but see Albert et al., 2010; Anderegg et al., 2018). Third, we need to clarify if and how plant (“soft”) traits used classically to describe ecological strategies are connected to plant metabolism and physiology (“hard” traits).

The development of near-infrared spectroscopy (NIRS) has provided a unique, fast, and reliable tool enabling the collection of a myriad of traits non-destructively (Foley et al., 1998; Cozzolino et al., 2001; Pasquini, 2018; Silva-Perez et al., 2018). NIRS measures the light reflected from a sample after irradiating it with wavelengths ranging from visible (VIS, 400–700 nm), near-infrared (NIR, 700–1,100 nm), to shortwave infrared (SWIR, 1100–2,500 nm). This provides a signature of the physical and chemical characteristics of the sample (Box 1). NIRS has been widely used for determining chemical traits in various fields. For instance, it is extensively used to characterize chemical products in pharmaceutical, agricultural, and food sectors (Shepherd and Walsh, 2007; Wójcicki, 2015; Biancolillo and Marini, 2018; Pasquini, 2018). In plant science, NIRS takes an increasingly important place as a high-throughput, cost-efficient method for the characterization of biodiversity (Arslan et al., 2018; Silva-Perez et al., 2018; Burnett et al., 2021; Kothari et al., 2021). For instance, it is widely used to predict differences in leaf palatability, digestibility, and decomposability—through lignin and fiber content—between species (Birth and Hecht, 1987; Andrés et al., 2005). The advantages of this method are numerous: spectral measurements are extremely rapid, taking only a few seconds, a single spectral measurement simultaneously captures multiple diverse plant traits (Petit Bon et al., 2020), minimal or no sample preparation is required, and the measurements are non-destructive which allows to track trait changes over time and avoids interfering with the organism.

BOX 1 **Principle of near−infrared spectroscopy (NIRS) for plant characterization**

The leaf spectral reflectance is based on the low reflectivity in the visible part of the spectrum (400–700 nm), due to a strong absorption by photosynthetic pigments, and the high reflectivity in the near infrared (700–1,100 nm) produced by a high scattering of light by the leaf mesophyll tissues (Knipling, 1970). For instance, in the SWIR part of the spectrum (1100–2,500 nm), the reflectance intensity is affected by the water, cellulose, protein, and lignin content of plant tissues (Rascher et al., 2010). Healthy leaves emit radiation in the thermal infrared band (≈10 μm) according to their temperature, because of their high water content (emissivity between 0.97 and 0.99). The leaves appear green because the green light band (550 nm) is reflected relatively efficiently when compared with the blue, yellow, and red bands, which are absorbed by photoactive pigments. This absorption at different wavelength produces a spectrum of light reflectance (Figure I), which can be treated as a “signal” of the leaf physical and chemical properties.

**Figure I:**
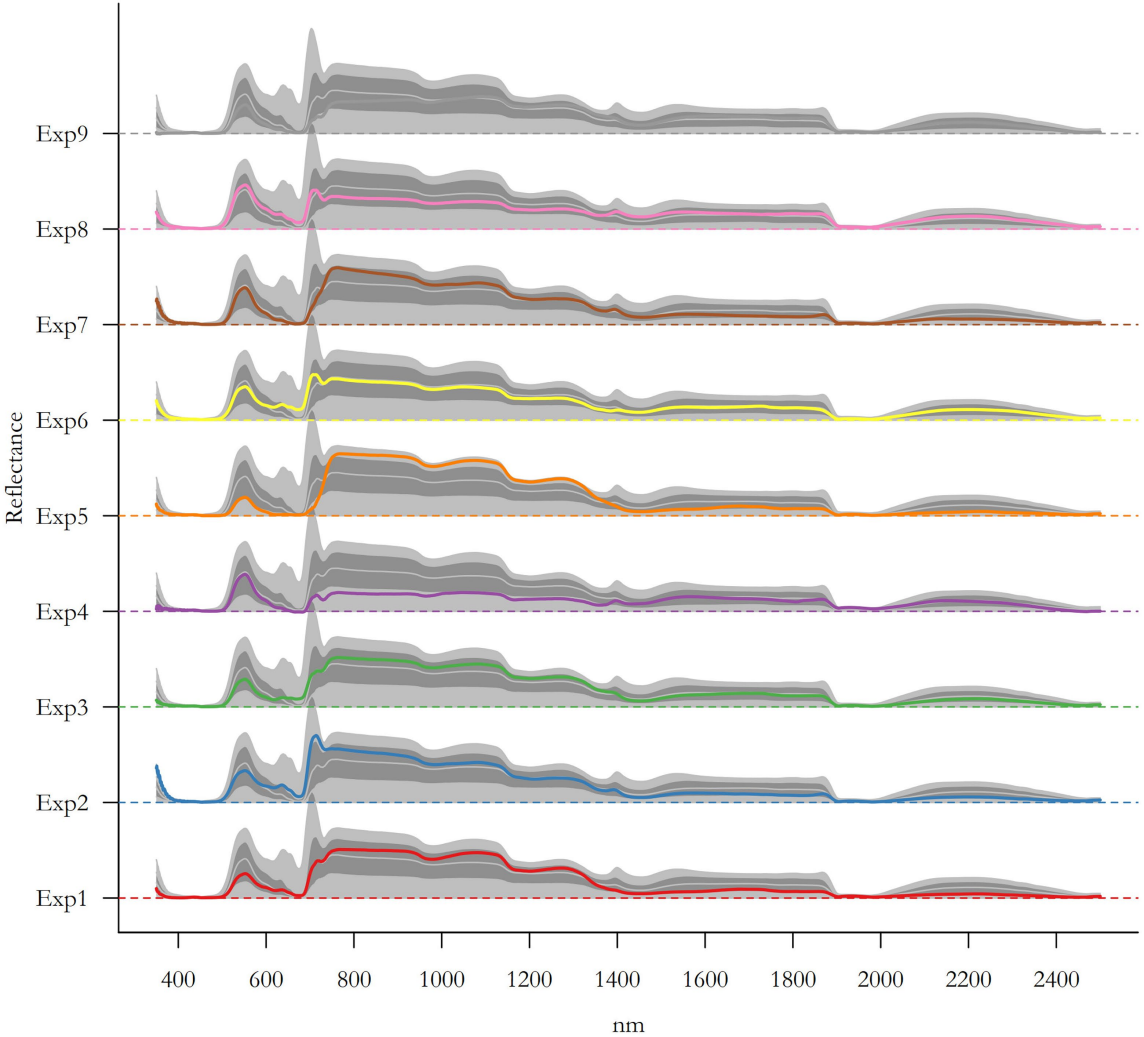
Leaf reflectance as a function of light wavelength. All spectrum available in the database used to analyze the ability of spectral reflectance to predict trait values and plant categories are represented here and colored according to the experiment they come from (see Supplementary Table S1 and Supplementary Material for details about experiments, conditions, as well as number of spectra per experiment). Colored lines represent the mean absorbance spectra, light grey lines represent the median absorbance spectra, dark shaded area represents spectra with absorbance ranging between the 5 and 95th percentiles, and light shaded area represents the entire absorbance range covered by the spectra.

The physical association between leaf properties and light reflectance is particularly useful to investigate leaf composition, functioning, and diversity. Different leaves will have different spectral signatures depending on their structure and chemical composition. For example, leaf nitrogen concentrations are associated with wavelengths absorbed by chlorophyll a and b in the visible part of the spectrum (400–700 nm), the spectral red edge (700–760 nm), and proteins in the SWIR (1,300–2,500 nm; Gitelson and Merzlyak, 1997; Kokaly, 2001). In the SWIR (SWIR; 700–1,300 nm), structures such as palisade cell density are important determinants of the spectral reflectance because of the very low effective photon penetration distance at these wavelengths.

While NIRS data are simple to acquire and rapidly generate a very large amount of information, they also require extensive post-processing, *via* chemiometric and multivariate statistical analyses. Usually, spectral information can be exploited through the development of calibration models relating spectra and reference trait data. Calibration models are built with a representative subsample of a complete data set, in terms of the range of spectral variation treated (Foley et al., 1998). After building and validating models linking plant spectra to independently measured traits in the calibration dataset, the trait values of new samples are predicted from their spectra using these models. For that, different statistical methods are commonly used to predict trait data from spectra, including partial least squares regression (PLSR; Wold et al., 1983), principal components analysis (Dreccer et al., 2014), and 2D correlation plots (Darvishzadeh et al., 2008). However, the performance of these methods, and especially PLSR, in estimating plant traits has been shown to vary significantly across species and growth conditions (Fu et al., 2020). In recent years, machine learning approaches have become widespread in multiple fields due to their better predictive performance. Machine learning and more particularly deep learning techniques—specific machine learning algorithms using a series of neural networks (Box 2)—are promising methods to improve the statistical analysis of high-throughput data (Mishra and Passos, 2021).

BOX 2 **The promise of deep learning to analyze NIRS**

Chemometrics, the science of extracting information from chemical systems, faces multiple challenges while studying the link between samples near infrared spectra and desired traits. Partial least square regression (PLSR), the reference method, is well designed to address many of these but still exhibit some weakness that native properties and tools associated with deep learning may allow to tackle.

First, the inherent and unwanted variability associated with the spectrometric measure result in a highly noisy signal (e.g., environmental, machine, or sample variability). To deal with it, reference methods rely on pretreatment (i.e., filtering) of the signal and removal of the spectral outliers. Pretreatment works by removing noise and linearizing the response of a variable. As the number of possible pretreatments increases with research progress, finding the optimal solution empirically becomes more and more a challenge. Moreover, pretreatment development is optimized for its suitability to filter spectra in combination with a reference model (i.e., PLSR in most situations). This may result in suboptimal solution and loss of information. Conversely, some deep learning algorithms are particularly efficient in filtering input signals. Cui and Fearn (2018) illustrated how the convolutional layer can continually tune the variables in the filter, until it finds the best form of preprocessing. This means the spectroscopic preprocessing done by the convolutional layer is more flexible and saves a lot of effort when building new calibrations. In addition, because PLSR is highly sensitive to outliers, a common technique aims to remove spectra based on distance metrics and arbitrary threshold (Wadoux et al., 2021). This normative procedure could lead to the loss of informative spectra, particularly while dealing with small datasets.

In deep learning, many techniques (e.g., robust loss function and early stopping) allow us to deal with noise. This includes original signal noise but also artificially added noise allowing for data augmentation and regularization in order to minimize overfitting and increase robustness. Deep learning allows keeping all spectra without questioning the representativeness of a highly complex and spatially explicit signal (i.e., the spectra) based on a simple global distance.

Second, wavelength range and resolution of the actual spectrometer allow for a highly multivariate signal. Reference methods to deal with this often imply dimension reduction (e.g., PLSR), leading to a loss of information. The ability of multilayer networks trained with gradient descent to learn complex high dimensional non-linear mappings makes them obvious candidates. Indeed, deep networks proved to have the theoretical guarantee that they can avoid the curse of dimensionality for many problems (Poggio et al., 2017). Among deep learning algorithms, convolutional neural networks (CNN) are known to efficiently take care of variable selection. And as already mentioned, deep learning algorithms come with multiple useful techniques to deal with the overfitting risk (e.g., batch normalization, dropout, early stopping, and noise generation).

Finally, one of the main challenges for chemometrics is to infer traits values based on the reflectance of a limited number of chemical bonds that may be shared by multiple compounds (i.e., molecules). Therefore, the prediction of the compound’s content relies on a large number of very indirect relationships between reflectance values. This is already true for chemical composition (e.g., most carbohydrates exhibit only limited differences in their chemical composition) but it is even more obvious while working on functional traits resulting from multiple physiochemical changes. Moreover, the functional properties potentially arise from non-linear relationship or threshold, compensatory, moderator, and mediator effects at tissue, molecule, and chemical bounds scales. These multiscale non-linear relationships are hardly modeled by reference methods such as partial least squares regression (PLSR). The multilayer nature of deep learning algorithms allows for the identification of multiscale patterns and easily tackles non-linearity.

Spectral predictions of functional traits have been used to screen interspecific diversity across individual leaves, canopies, and biomes (Doughty et al., 2011; Roelofsen et al., 2014; Serbin et al., 2016; Wu et al., 2016; Chavana-Bryant et al., 2017). Yet, investigating intraspecific variability is crucial to connect global trait diversity to the underlying mechanisms of selection, genetic differentiation, and evolutionary adaptation (Violle et al., 2014). In this context, the model species *Arabidopsis thaliana* is an interesting model to test the predictive power of plant diversity with NIRS. Indeed, this species exhibits a large range of functional trait variation across its geographic range (Lasky et al., 2012; May et al., 2017; Price et al., 2018; Takou et al., 2018; Sartori et al., 2019), and hundreds of natural ecotypes have been fully sequenced to examine the genetic determinism of this variation (1001 Genomes Consortium, 2016). Ecological studies have taken advantage of this feature to examine the evolution of plant strategies in response to climate (Price et al., 2018; Vasseur et al., 2018a,b; Exposito-Alonso et al., 2019; Exposito-Alonso, 2020; Lorts and Lasky, 2020). In addition, this model species has been widely used to examine metabolic and physiological features (Chan et al., 2010; Tohge et al., 2018; Wu et al., 2018). Advanced molecular techniques—“omics” approaches—allow accurate quantification of transcriptome, proteome, metabolome profiles, and fluxome (Beale et al., 2016).

On the one hand, we need to increase sample size across species, genotypes, and environments to obtain sufficient statistical power for broad generalization and predictions. On the other hand, the time-consuming careful methods required to measure physiologically meaningful (“hard”) traits limit studies to small sample sizes. We argue that a promising avenue to avoid this trade-off between generality and feasibility is to combine NIRS and deep learning computation. In this perspective article, we document how NIRS and deep learning paves the way for a quick and accurate quantification of plant trait diversity, ecological strategies, and physiological adaptation. In addition to examples from the literature, we compiled 21,032 spectra and 108 trait measurements from published and unpublished datasets (Supplementary Table S1) across 5,683 *Arabidopsis thaliana* plants grown in various conditions. Using this database and examples from the literature, we first show that NIRS can accurately predict leaf functional traits and identify major plant ecological strategies. Second, we show that NIRS predicts the growth conditions and the plant phenotypic response to stress. Finally, we provide evidence that NIRS can give access to new traits and functions, notably those related to plant life history, physiology, and metabolism.

## NIRS Quantifies Functional Trait Variability and Summarizes Plant Ecological Strategies

A key goal of trait-based ecology is to determine the physiological mechanisms of plant adaptation to the environment through the measurement of multiple traits related to resource-use, growth, development, and phenology. Recent efforts based on analyzing interspecific trait diversity have revealed functional tradeoffs at both local and global scales (Messier et al., 2016), which suggests that plant diversity is shaped by universal constraints. For instance, Díaz et al. (2016) recently analyzed more than 45,000 plant species and demonstrated that their diversity falls along two main phenotypic dimensions: one related to plant size, which affects competitive ability and dispersal; the other related to leaf anatomy, chemical composition, and longevity. This second phenotypic dimension, called the leaf economics spectrum (Wright et al., 2004), trades off traits positively related to nutrient retention—such as leaf dry matter content (LDMC), leaf nitrogen content (LNC), and leaf life span—with traits positively related to carbon acquisition—such as specific leaf area (SLA) and leaf photosynthetic rate. Importantly, the same trade-off has been observed within species (Vasseur et al., 2012; Anderegg et al., 2018; Sartori et al., 2019).

Different theories have been proposed to categorize plant phenotypic diversity into ecological strategies related to plant adaptation to the environment. Among these theories, Grime (1974) proposed that the quantitative variation in plant strategies is expected to result from their adaptation to contrasting levels of resource availability and disturbance. Following this hypothesis, plant strategies can thus be classified through a combination of three main axes, competitors (C), stress-tolerators (S), and ruderals (R; Grime, 1977, 1988). The “CSR” model suggests that the evolution of plant strategies is driven by trade-offs between resource capture and conservation, space occupancy, longevity, and dispersal. For instance, C-type plants invest resources into the growth of large organs to outcompete neighbors, S-type plants invest resources to conserve nutrients and protect tissues from stress damages, while R-type plants invest resources into rapid reproduction and propagule dispersal in highly disturbed environments. The CSR strategies are often depicted in a triangle with the primary types occupying the corners and intermediate forms, composed of a combination of types (e.g., “SR” and “CS”), arrayed within the triangle. The quantitative variations between CSR strategies are expected to result from plant adaptation to contrasting levels of abiotic stresses and disturbance. CSR variation has also been reported within species—notably in *A. thaliana*—and explained by evolutionary adaptation to the environment (May et al., 2017; Vasseur et al., 2018b). However, measuring through destructive methods, the numerous traits that enable the quantification of ecological strategies within—and *a fortiori* across—species remains an obstacle for the large-scale analysis of plant populations, which therefore limits our ability to temporally follow the relationships between plant traits, strategies, and the environment.

**Figure 1:**
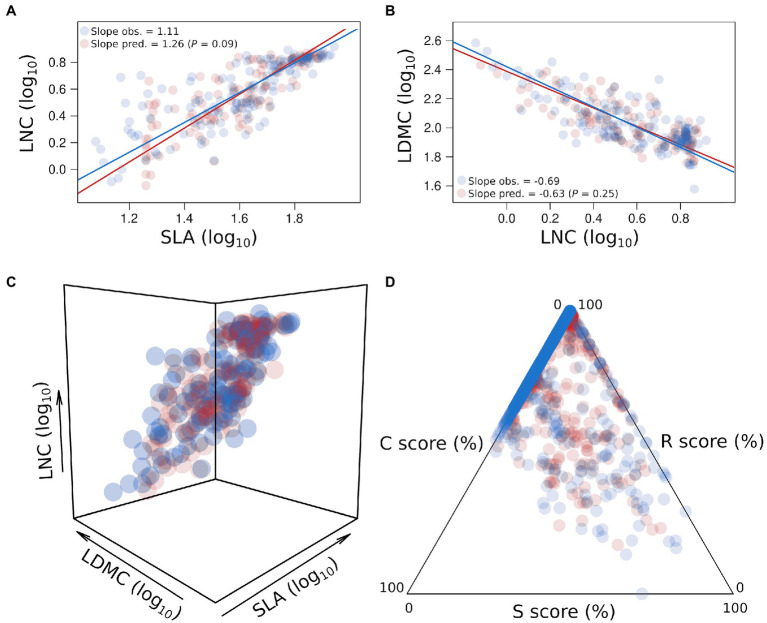
Predictions of the leaf economics spectrum and CSR strategies. Log_10_ relationships between specific leaf area (SLA, mm^2^ mg^−1^) and leaf nitrogen content (LNC, %; **A**); between leaf nitrogen content (LNC, %) and leaf dry matter content (LDMC, mg g^−1^; **B**). Only predicted values in the validation dataset (1/4 of the whole dataset, *n* = 123) were plotted here. Observed trait values are colored in blue and predicted trait values are colored in red. Regression lines have been estimated by standard major axis (SMA). *P* is the *p* value of the SMA test of slope difference between observed and predicted relationships. **(C)** 3D representation of the leaf economics spectrum between observed and predicted trait values in the validation dataset (*n* = 123). **(D)** CSR triangle between observed and predicted trait values in the validation dataset (*n* = 699) depicting the variation of plant ecological strategies between competitive ability (C), stress-tolerance (S), and ruderalism (R). CSR scores (%) have been measured from leaf traits following the method from Pierce et al. (2017) (see Supplementary Material). Only measurements performed on fully expanded but non-senescing leaves, and only under non stressing conditions, were used here.

Using convolutional neural network (CNN; Box 2, Supplementary Material, Supplementary Table S2) to analyze our database of spectra and traits in *A. thaliana*, we show that most leaf traits were accurately predicted (Table 1). For instance, only leaf relative water content (RWC) and the leaf isotopic ratio of nitrogen (δ^15^N) had validation *R*^2^ below 0.65 (Table 1). Yet, previous studies showed that δ^15^N can be predicted with NIRS (Kleinebecker et al., 2009). Here, correlations between measured and predicted values were the highest for leaf traits associated with the leaf economics spectrum (SLA, LDMC, and LNC, all *r*^2^ > 0.85; Table 1). Importantly, for these traits, the correlations calculated from the predicted data were the same as those calculated from the direct measurements (*p* > 0.05; Figures 1A–C). Previous studies showed that SLA can be accurately measured with NIRS from the level of individual leaves to the level of the tree canopies (Curran, 1989; Lymburner et al., 2000; Asner and Martin, 2008; Asner et al., 2009; Jacquemoud et al., 2009; Kokaly et al., 2009; Ecarnot et al., 2013; Singh et al., 2015; Serbin et al., 2016). Other LES traits have been shown to be well predicted by NIRS (Ecarnot et al., 2013; Kattenborn et al., 2017, 2019). In addition, LNC, another LES trait, can also be predicted using light reflectance at the individual leaf and at canopy levels (Sims and Gamon, 2002). Other traits related to resource-use and conservation can be predicted with spectroscopy, such as leaf age and photosynthetic capacity (Doughty et al., 2011; Chavana-Bryant et al., 2017). Thus, NIRS can provide estimates of integrated properties, such as trait covariations, whole-plant traits, and strategies. Accordingly, traits such as plant growth rate and water use efficiency (estimated by δ^13^C; Farquhar et al., 1989) were also well predicted in *A. thaliana* (*r*^2^ = 0.53 and 0.83, respectively; Table 1). By contrast, predictive performance was lower for plant life span here (*r*^2^ = 0.17), although previous studies showed that spectral profiles are able to capture key differences in plant life history (Ustin et al., 2004). Plant ecological strategies depicted by CSR scores were highly predicted in our database (Table 1; Figure 1D), as were CSR intermediate categories (e.g., SR, R/SR, S/SC, and CS), with a prediction accuracy estimated at 70% (Table 2).

**Table 1:** Prediction accuracy for functional traits.

Variable	*n*	Calibration	Validation				
		*SD*	*R* ^2^	RMSE	Bias	Slope	RPD
LDMC (mg g^−1^)	2,932	52.73	0.86	16.10	0.38	1.06	3.28
SLA (mm^2^ mg^−1^)	3,423	20.90	0.85	7.47	0.14	1.01	2.80
LNC (%)	1,961	2.18	0.93	0.53	−0.06	0.97	4.12
Leaf thickness (μm)	4,143	178.08	0.89	69.49	2.79	1.02	2.56
RWC (%)	1,421	22.06	0.17	4.52	0.40	1.27	4.88
LCC (%)	1,960	4.78	0.65	1.17	0.03	0.86	4.10
δ^13^C	1,222	1.59	0.83	0.62	−0.04	0.95	2.56
δ^15^N	1,223	3.76	0.28	1.83	−0.13	0.82	2.06
Plant lifespan (days)	1,403	10.55	0.17	8.01	−1.31	0.86	1.32
Plant growth rate (mg d^−1^)	701	0.01	0.53	0.00	0.00	0.96	1.94
*C* score (%)	2,905	10.25	0.88	3.28	−0.02	1.03	3.13
*S* score (%)	2,905	11.64	0.75	2.57	0.19	1.11	4.53
*R* score (%)	2,905	17.03	0.87	4.79	0.33	0.99	3.55

LDMC, leaf dry matter content; SLA, specific leaf area; LNC, leaf nitrogen content; RWC, relative water content; LCC, leaf carbon content; δ^13^C, fraction of ^13^C isotope; and δ^15^N, fraction of ^15^N isotope. CSR scores were estimated from leaf traits by the algorithm from Pierce et al. (2017). *n* is the total number of leaves used for modelling from our database that are associated with both trait and spectra measurements. All predictions have been obtained from convolutional neural network (CNN) models (see Supplementary Material for details). SD, standard deviation; RMSE, root mean square deviation; and RPD, relative percent difference.

**Table 2:** Prediction accuracy for five plant categories.

	Calibration accuracy (%)	Validation accuracy (%)
Survival (2)	0.988	0.915
Genotypes (10)	0.831	0.640
Indoor/Outdoor (2)	0.998	1.000
CSR categories (11)	0.980	0.700
Treatment (2)	0.955	0.714

Plant survival has two categories (dead or alive), which were measured according to the protocol described in Estarague et al. (2021). Genotypes have 10 categories corresponding to the 10 natural accessions used here. Indoor/outdoor represents whether a plant has been grown in a greenhouse or growth chamber (indoor) or in a common garden (outdoor) across all the experiments included in the database used here. CSR categories are the intermediate CSR classes estimated from leaf traits by the algorithm from Pierce et al. (2017), such as R/SR, S/SC, RS, and C/CSR (see Supplementary Material). Treatment has two categories (control and water stress) from the dedicated experiments included in the database (see Supplementary Material). All predictions have been obtained from CNN models.

Consistent with previous studies (Le, 2020; Barradas et al., 2021; Mishra and Passos, 2021), our results show that NIRS coupled with deep learning is a powerful tool to assess phenotypic variations in plants. Using 15 functional and metabolomic traits, we show that deep learning methods outperform classical analytical techniques such as PLSR (Supplementary Table S3). Moreover, deep learning approaches have numerous advantages compared to PLSR (Box 2). In particular, it does not require preprocessing of the data (cleaning and standardization of the spectra and removal of outliers), which often depends on the user’s choice and differs from one dataset to another. Importantly, analyzing the spectral signature of plants with deep learning allows determining with reasonable accuracy the plant genotype. For instance here, genotype identity was correctly predicted for 64% of the tested accessions (Table 2), as previously observed in maize (Rincent et al., 2018). Such estimation opens promising avenues as an alternative to expensive sequencing technologies, as well as to combine genomics with phenomics.

## Measuring Plant Responses to the Environment With NIRS

Large-scale comparisons of ecological strategies have been performed with large databases of trait values measured on many species under various conditions, from lab benches to greenhouse, common garden, and field conditions (Kattge et al., 2020). Although these trait databases are used to interpret plant adaptation to the environment, they surprisingly contain very little information about the response of the measured plant properties (demographics, growth rate, and traits) to the environment (Salguero-Gómez et al., 2018). Indeed, comparative approaches generally focus on interspecific variation, considering a mean trait value per species and neglecting intraspecific variability and phenotypic plasticity (but see Albert et al., 2010, 2011). For instance, CSR strategies, which should reflect environmental specialization and specific stress resistance, still remain largely unconnected to the plant evolutionary responses to biotic and abiotic stresses (Takou et al., 2018).

Spectral measurements are widely used to design screening protocols for plant drought responses (Shepherd and Walsh, 2007; Barradas et al., 2021; Burnett et al., 2021). For example, Cabrera-Bosquet et al. (2011) used spectra to accurately predict genotypic differences in the kernel and leaf water content in maize grown under different water treatments. In addition, spectral measurement is a promising method for detecting the severity of damage caused by pathogens, especially for those leaving no visible signs (Spinelli et al., 2004; Sabatier and Rutherford, 2013). Indeed, healthy plants interact (absorb, reflect, emit, transmit, and fluoresce) with electromagnetic radiation in a manner different from that of infected or damaged plants (Li et al., 2014).

**Figure 2:**
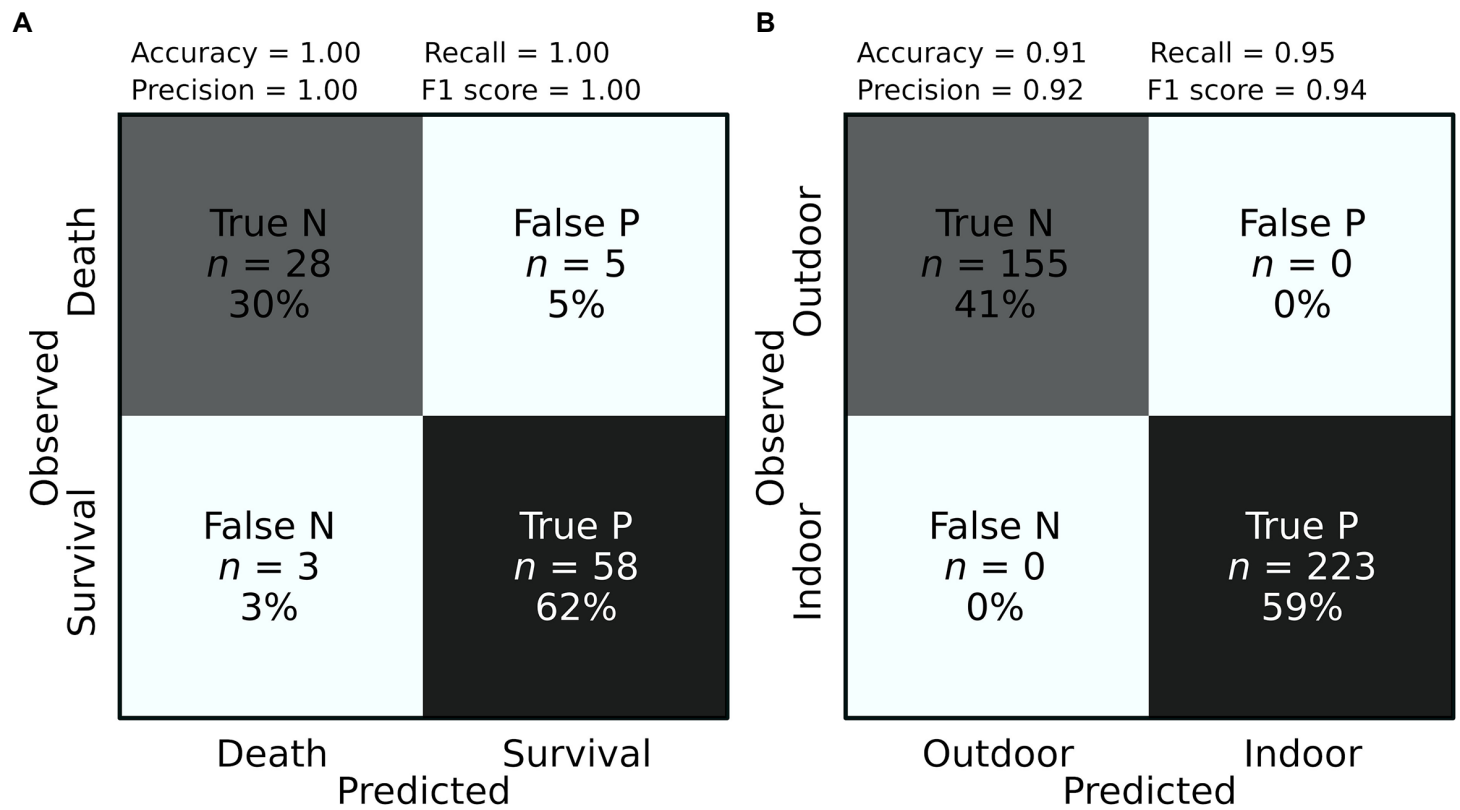
Prediction accuracy of plant survival and growth conditions. Confusion matrices showing the classification performance for the prediction of **(A)** plant survival (positive P) and mortality (negative N), and **(B)** the growth condition: indoor (positive P) vs. outdoor (negative N). Precision score = true P/(false P + true P). Recall score = true P/(false N + true P). Accuracy Score = (true P + true N)/(true P + false N + true N + false P). F1 Score = 2*Precision score*Recall score/(Precision score + Recall score).

To further explore the potential of NIRS as a predictive tool of plant stress level, we used experimental data included in our database (Supplementary Material) from 30 genotypes of *Arabidopsis thaliana* subjected to water deficit combined with either high or low (freezing) temperatures (Estarague et al., 2021). All plant individuals were measured for leaf NIRS in the course of the treatment, and survival was visually recorded after the treatment. Both measured and CNN-predicted survival rates varied from 14 to 80% depending on the genotype, with an estimated accuracy of survival prediction of 91% in an external validation dataset (Table 2; Figure 2A). Importantly, spectral measurements were taken during the treatment, before individuals started showing visible signs of death (Estarague et al., 2021). This suggests that NIRS is a powerful tool to estimate stress effects leading to plant death early on, even before any visible sign of adverse effects.

Convolutional neural network models were able to accurately predict the environmental treatment in which plants were grown (control vs. water stress; prediction accuracy = 71%, Table 2). More surprisingly, CNN models reached 100% accuracy to predict if a plant was grown indoor (growth chamber and greenhouse) vs. outdoor (common garden; Figure 2B; Table 2). This result not only demonstrates the capacity of NIRS and deep learning to characterize the environmental conditions in which plants are cultivated but also suggests that plants grown indoor and outdoor have very contrasted spectral signatures. In turn, these questions our ability to draw conclusions about plant adaptation in natural conditions from experiments led in controlled conditions (growth chamber and greenhouse).

## Metabolomics as a New Phenotypic Dimension: Future Perspectives for the Characterization of Plant Ecological Strategies

A broader screening of the metabolic pathways involved in the physiological adaptation of plants to contrasting environments is a promising avenue for ecology in the future. So far, large comparative approaches remain limited by the type and availability of traits collected from the literature and organized into shared databases (Kattge et al., 2020). This constraint reduces our ability to fully understand the drivers of phenotypic diversity, as well as to identify new and ecologically meaningful axes of phenotypic variation. In this perspective, NIRS allows us to detect a large variety of commonly measured chemical compounds such as phosphorus (P)—a key element of the leaf economics spectrum— and base cations [calcium (Ca), potassium (K), and magnesium (Mg)], and other micronutrients (Cozzolino et al., 2001; Ortiz-Monasterio et al., 2007; Galvez-Sola et al., 2015; Ercioglu et al., 2018; de Oliveira et al., 2019; Yu et al., 2019; Prananto et al., 2021). This opens new avenues to link resource-use strategies with plant elemental composition, fluxes, stoichiometry, and beyond, with nutrient cycling in ecosystems (Ustin et al., 2004). In addition, studies have shown that not only LNC but also chlorophyll *a* and *b* can be predicted using reflectance and transmittance of light from individual leaves and at canopy level (Sims and Gamon, 2002).

Using quantitative measurements of 67 metabolites with GC–MS and LC–MS (Supplementary Material), we investigated whether NIRS can estimate variations in foliar content of sugars (e.g., glucose and fructose), hormones (e.g., salicylic acid, auxin, and abcissic acid), and secondary metabolites (e.g., phenolic compounds and glucosinolates). Our results show that prediction accuracy (estimated in an external dataset; Supplementary Material) was highly variable between metabolites. For instance, validation *r*^2^ ranged from 0% for the poorest predictions (see examples in Table 3) to 85% for the highest (dihydro caffeoyl glucuronide; Table 3). For sugars, the best predictions were obtained for fructose, cellobiose, mannose, and raffinose (Table 3). Among hormones, only auxin (IAA) and jasmonic acid (JA) were satisfactorily predicted by NIRS (Table 3), while other hormones were very poorly predicted (for instance, *r*^2^ < 0.10). Glucosinolates are a class of metabolites produced by the Brassicaceae family, which are involved in plant defense against herbivores (Ratzka et al., 2002). Many of them showed relatively high prediction accuracy (e.g., glucoraphenin and neoglucobrassicin with *r*^2^ > 0.70; Table 3), which paves the way for predicting plant responses to herbivore attack on many individuals at low cost. Finally, many other secondary metabolites showed substantial prediction accuracy (e.g., *r*^2^ > 50%; Table 3), although prediction accuracy was very variable between metabolites. More studies are needed to fully explore the potential of NIRS and deep learning to predict leaf chemistry and metabolisms. However, applying NIRS—coupled with deep learning computation—for high-throughput phenotyping of plants from cellular level to whole-plant level is perhaps the most exciting perspective of this approach.

**Table 3:** Prediction accuracy for 67 metabolites.

	Variable	Caibration validation					
		*SD*	*R* ^2^	RMSE	Bias	Slope	RPD	
Sugars	Glucose	6764.56	0.14	1621.88	−4.49	0.95	4.17	
	Fructose	10240.92	0.56	1316.93	352.08	1.17	7.78
	Sucrose	11380.72	0.00	2086.69	538.48	−12.55	5.45
	Fucose	28.65	0.03	1.90	0.37	0.75	15.04
	Isomaltose	26.02	0.16	6.58	1.44	1.41	3.95
	Cellobiose	157.51	0.39	73.21	19.87	1.85	2.15
	Arabinose	37.57	0.00	51.42	9.39	100.65	0.73
	Galactose	293.66	0.18	304.29	82.21	1.11	0.97
	Inositol	911.06	0.31	136.28	23.17	1.29	6.69
	Maltose	58.40	0.02	57.31	19.37	0.86	1.02
	Mannose	219.79	0.42	35.78	12.77	2.19	6.14
	Raffinose	644.65	0.57	457.00	112.77	1.12	1.41
	Rhamnose	68.56	0.02	95.56	17.09	−1150.74	0.72
	Ribose	32.35	0.00	42.17	13.41	138.61	0.77
	Palatinose	236.89	0.00	294.60	36.80	−5.60	0.80
	Melezitose	15.62	0.38	7.47	1.31	1.26	2.09
	Melibiose	200.00	0.09	264.69	47.47	0.69	0.76
	Trehalose	176.00	0.00	146.34	23.78	−1.69	1.20
	Xylose	35.75	0.13	7.09	1.54	1.32	5.04
Hormones	ABA	12.54	0.06	11.25	1.43	0.57	1.12	
	IAA	21.37	0.26	18.16	1.84	0.95	1.18
	JA	337.70	0.29	197.91	31.53	1.03	1.71
	SA	799.00	0.00	495.41	147.44	−10.54	1.61
	CMLX	7277.61	0.02	8086.67	2421.27	63.66	0.90
Glucosinolates	Glucoalysiin	28.79	0.10	27.76	3.95	1.05	1.04		
	Glucobrassicin	1462.69	0.15	914.32	210.01	0.76	1.60	
	Glucoerucin	12.22	0.39	5.88	0.51	0.86	2.08	
	Gluconapin	5005.90	0.00	4703.53	2123.30	0.43	1.06	
	Gluconasturtiin	94.36	0.00	91.73	12.46	0.63	1.03	
	Glucoraphanin	1308.98	0.00	1166.48	250.14	0.22	1.12	
	Glucoraphenin	1.78	0.74	0.62	0.07	0.91	2.88	
	Epigallocatechin	210.86	0.27	163.05	2.91	0.83	1.29	
	Progoitrin	666.26	0.01	564.65	135.83	0.38	1.18	
	Epiprogoitrin	6316.22	0.09	5944.42	1814.64	0.74	1.06	
	Isobutyl	473.57	0.03	356.50	56.56	0.67	1.33	
	Glucosinalbin	10.35	0.00	7.96	1.28	2.52	1.30	
	Sinigrin	4445.20	0.07	4259.39	1571.86	1.04	1.04	
	Hexyl	49.96	0.00	45.61	12.28	0.53	1.10	
	Butyl	5.49	0.51	3.20	−0.24	1.07	1.72	
	Neoglucobrassicin Peak1	265.97	0.73	273.80	59.08	1.86	0.97	
	Neoglucobrassicin Peak2	1051.25	0.06	254.92	24.16	0.41	4.12	
	X3MTP	47.48	0.51	9.63	0.36	1.41	4.93	
	X5MTP	20.76	0.61	11.56	1.14	1.40	1.80	
	X6MSH	51.83	0.22	48.64	9.55	1.09	1.07	
	X7MSH	261.68	0.18	277.93	88.23	1.19	0.94	
	X7MTH	244.30	0.36	224.81	36.56	1.04	1.09	
	X8MSO	2013.33	0.31	1528.42	169.92	0.87	1.32	
	X8MTO	1278.38	0.17	1053.50	176.17	0.85	1.21
Other secondary metabolites	Apigenin rutinoside	1140.31	0.31	848.50	73.33	0.63	1.34		
	Caffeic Acid	30.01	0.32	0.96	−0.20	0.74	31.31	
	Chlorogenic Acid	29.55	0.66	16.29	1.38	1.09	1.81	
	Citrat	2647.54	0.44	1894.98	169.09	1.08	1.40	
	Cyanidin rhamnoside	1431.34	0.53	842.46	−56.16	0.81	1.70	
	Cyanidin sophorosid glucoside	674.85	0.31	387.08	88.61	1.04	1.74	
	Dihydro caffeoyl glucuronide	27.05	0.85	8.96	0.01	1.12	3.02	
	Fumarat	294.76	0.10	174.41	18.17	0.68	1.69	
	Kaempherol glucosyl rhamnosyl glucoside	989.20	0.14	518.91	97.70	0.69	1.91	
	Kaempherol rutinoside	2788.98	0.59	1613.31	127.58	0.88	1.73	
	Kaempherol xylosyl rhamnoside	1362.13	0.56	774.66	7.04	0.88	1.76	
	Malat	1078.18	0.16	786.53	133.47	0.61	1.37	
	m-Coumaric Acid	144.26	0.00	143.67	18.09	0.84	1.00	
	p-Coumaric Acid	4.00	0.46	1.35	−0.08	1.02	2.95	
	Pelargonidin cumaroyl diglucoside glucoside	69.47	0.65	34.69	−0.28	0.94	2.00	
	Pelargonidin sambubioside	291.72	0.47	223.17	13.31	0.81	1.31	
	Prenyl naringenin	36.74	0.63	14.89	−2.09	0.93	2.47	
	Quercetin glucoside	56.73	0.23	54.09	11.77	1.41	1.05	
	Succinat	60.74	0.16	45.15	0.70	0.93	1.35

Metabolites have been measured with GC–MS or LC–MS depending on the metabolite (*n* = 124 per metabolite) on leaves harvested on 4-week old plants grown in the greenhouse. Sugars are given in μmol/gFW; hormones in ng/gFW. For glucosinolates and other secondary metabolites, foliar relative concentrations were estimated by dividing the peak area corresponding to the metabolite by the fresh weight of the sample. SD, standard deviation; RMSE, root mean square deviation; and RPD, relative percent difference. All predictions have been obtained from CNN models (see Supplementary Material for details).

## Conclusion

In this paper, we argue that NIRS coupled with recent advances in deep learning approaches is a promising method to broadly capture various information about plant functioning, ecological strategies, response to environment, and metabolism. In particular, NIRS affords considerable time and cost savings (spectrum acquisition lasts only a few seconds), and without using hazardous chemicals. In addition, samples can be analyzed in neither their natural form without destruction nor any special sample preparation. Thus, NIRS makes it possible to create extensive databases of traits at different temporal, spatial, and taxonomic scales and facilitate the adoption of phenomics into ecology. It might provide a reliable tool for the characterization of plant populations across geographical ranges, specifically if combined with other omics approaches and deep learning computation. Of course, developing calibration equations takes time, but selecting a suitable subset of samples to use in the calibration equation and validating the calibration equation take only a matter of hours in addition to standard laboratory work to chemically analyze the subset. Clearly, NIRS is more suited for larger data sets than those containing only a few samples. As calibration equations keep available for future studies, the time and financial cost of calibrations will decrease. Thus, adopting NIRS in trait-based ecology would literally multiply the number of species, genotypes, and environments potentially measurable, a key point to link functional trait variation to plant physiology and adaptation.

## Data Availability Statement

The raw data supporting the conclusions of this article will be made available by the authors, without undue reservation.

## Author Contributions

FV led the writing of the manuscript. All authors contributed critically to the drafts and gave final approval for publication.

## Funding

This work was supported by INRAE, CNRS, the French Agency for Research (ANR grant ANR-17-CE02-0018-01; “AraBreed” to FV, DV, and CV), Région Occitanie (FEDER FSE IEJ 2014-2020; Project PHENOPSIS 2.0), and the European Research Council (ERC;“CONSTRAINTS”: grant ERC-StG-2014-639706-CONSTRAINTS to CV). Metabolite analytics were funded by the DeutscheForschungsgemeinschaft (DFG, German Research foundation) – Projektnummer INST 37/696-1 FUGG.

## Conflict of Interest

The authors declare that the research was conducted in the absence of any commercial or financial relationships that could be construed as a potential conflict of interest.

## Publisher’s Note

All claims expressed in this article are solely those of the authors and do not necessarily represent those of their affiliated organizations, or those of the publisher, the editors and the reviewers. Any product that may be evaluated in this article, or claim that may be made by its manufacturer, is not guaranteed or endorsed by the publisher.
