# Erratum: A perspective on plant phenomics: Coupling deep learning and near-infrared spectroscopy

**DOI:** 10.3389/fpls.2022.985970

**Published:** 2022-08-02

**Authors:** Frontiers Production Office

**Affiliations:** Frontiers Media SA, Lausanne, Switzerland

**Keywords:** *Arabidopsis thaliana*, near-infrared spectroscopy (NIRS), multivariate analysis, machine learning, functional traits, metabolomics, trait-based ecology

Due to a production error, affiliations 9 and 10 are incorrect.

For affiliation 9, instead of “^9^LEPSE, Univ Montpellier, INRAE, Institut Agro, Montpellier, France” it should be “^9^CEFE, Univ Montpellier, CNRS, EPHE, Institut Agro, IRD, Montpellier, France.”

For affiliation 10, instead of “^10^CEFE, Univ Montpellier, CNRS, EPHE, Institut Agro, IRD, Université Paul Valery Montpellier, Montpellier, France” it should be “^10^LEPSE, Univ Montpellier, INRAE, Institut Agro, Montpellier, France.”

In addition, there was an error regarding the affiliations for Justine Floret, Elena Kazakou, and Denis Vile. Instead of “Justine Floret^1,9^,” “Elena Kazakou^10^,” and “Denis Vile^9^,” it should be “Justine Floret^1,10^,” “Elena Kazakou^9^,” and “Denis Vile^10^.”

The publisher apologizes for this mistake. The original article has been updated.

