# Supplementary material for: A Perspective on Plant Phenomics: Coupling Deep Learning and Near-Infrared Spectroscopy
Source: Front Plant Sci. 2022 May 20;13:836488. doi: 10.3389/fpls.2022.836488 (PMC9163986; doi:10.3389/fpls.2022.836488)
Supplement: Supplementary file 1 [file Data_Sheet_1.pdf]

A perspective on plant phenomics: coupling deep learning and near-infrared spectroscopy

Francois Vasseur[1], Denis Cornet[2,3], Grégory Beurier[2,3], Julie Messier[4], Lauriane Rouan[2,3], Justine Bresson[1], Martin Ecarnot[3], Mark Stahl[5], Simon Heumos[6,7], Marianne Gérard[1], Hans Reijnen[1], Pascal Tillard[8], Benoît Lacombe[8], Amélie Emanuel[1,8], Justine Floret[1,10], Aurélien Estarague[1], Stefania Przybylska[1], Kevin Sartori[1], Lauren M. Gillespie[1], Etienne Baron[1], Elena Kazakou[9], Denis Vile[10], Cyrille Violle[1]

Affiliation:
[1] CEFE, Univ Montpellier, CNRS, EPHE, IRD, Montpellier, France
[2] CIRAD, UMR AGAP Institut, F-34398 Montpellier, France
[3] UMR AGAP Institut, Univ Montpellier, CIRAD, INRAE, Institut Agro, F-34398 Montpellier, France
[4] Department of Biology, University of Waterloo, 200 University Avenue West, Waterloo N2L 3G1 Canada
[5] Center for Plant Molecular Biology (ZMBP), University of Tübingen, Germany
[6] Quantitative Biology Center (QBiC), University of Tübingen, Tübingen, Germany
[7] Biomedical Data Science, Dept. of Computer Science, University of Tübingen, Tübingen, Germany
[8] BPMP, Univ Montpellier, CNRS, INRAE, Montpellier, France
[9] CEFE, Univ Montpellier, CNRS, EPHE, Institut Agro, IRD, Université Paul Valery Montpellier, Montpellier, France
[10] LEPSE, Univ Montpellier, INRAE, Institut Agro, Montpellier, France

Supplementary Information

Database of traits and spectra

We compiled phenotypic data and spectra from seven experiments with published (Sartori et al., 2018; Vasseur et al., 2018; Estarague et al., 2021; Sartori et al., 2022) and unpublished datasets. All these experiments were performed on the model species *Arabidopsis thaliana*. Some of them in controlled conditions (greenhouse or high-throughput phenotyping platform such as PHENOPSIS (Granier et al., 2006)), others in common gardens, and a few collected in the wild. In total, our database contains 21,032 spectra and 108 traits measured on 5,683 plants, which are summarized in Table S1.

Table S1. Summary of the experiments used in this study

idExp	Experiment name	IndOut	Condition	Treatment	Plant	Spectrum	Genotype	Individual	Traits
Exp1	AraBreed_Outside_Spring2018	Outdoor	Common garden	Control<br>Herbivory<br>Water stress<br>Water stress / herbivory	Flowering	1791	NA	591	104
Exp2	AraBreed_Outside_Spring2019	Outdoor	Common garden	Control<br>Herbivory<br>Water stress<br>Water stress / herbivory	Flowering	227	NA	227	7
Exp3	AraBreed_PHENOPSIS_2018	Indoor	Growth chamber	Control	Flowering	745	NA	702	13
Exp4	AraBreed_Pilot_2017	Indoor	Growth chamber	Control	Vegetative	313	11	59	60
Exp5	CEFE_2018_JulieM	Indoor	Greenhouse	Control	Bolting	114	29	62	9
Exp6	Herbivory_2015	Indoor	Greenhouse	Control<br>Herbivory	Bolting<br>Flowering	5331	211	1419	9
Exp7	PlastEdge_2019	Indoor	Greenhouse	Control<br>HightTemperature<br>LowTemperature<br>WaterStress/HightTemperature<br>WaterStress/LowTemperature	Vegetative	1646	30	1646	10
Exp8	Resorption_2017_KevinS	Indoor	Greenhouse	Control	Bolting<br>Flowering	10789	149	939	9
Exp9	TE_Outdoor_2017	Outdoor	Wild	NA	Bolting	76	NA	38	9
**TOTAL**	**9**	**2**	**4**	**8**	**3**	**21032**	**343**	**5683**	**108**

Spectrum acquisition

In all experiments, NIRS measurements were performed using a LabSpec 4, spectrometer (ASD Inc., Analytik Ltd, UK). Light absorbance of leaf tissues was recorded for the spectral region 350-2500 nm for one to six different points across the leaf, avoiding the midrib. Depending on the experiment, measurement were taken on a fully-expanded but non-senescing leaf, or a growing (non-mature) leaf, or either a senescing leaf (see Table S1 for further information). In the comparative analyses performed in this study (Figure 2, Tables 1, 3, and S3), only measurements performed on fully-expanded but non-senescing leaves, and only under non stressing conditions, were used.

Functional trait measurement

In all experiments included in the database, traits were measured following standardized protocols (Pérez-Harguindeguy et al., 2016), which are detailed in related references (Sartori et al., 2018; Vasseur et al., 2018; Estarague et al., 2021) and briefly described below.

Plant lifespan was measured in the 'PlastEdge' experiment (Estarague et al., 2021) as the time in days between sowing and the end of reproduction, when the first fruits become senescing. Plant growth rate was measured in the 'AraBreed-PHENOPSIS' experiment (unpublished). The total projected leaf area of the rosette (RA, cm^2) was determined every 2 to 3 days from zenithal images of the plants. A sigmoid curve was fitted for each plant following:

$$RA = \frac{a}{1+e^{\frac{d-d_0}{b}}} \qquad (1)$$

where *d* is the number of days after emergence of the firsts two true leaves, *a* is the maximum vegetative rosette area, d_0 is the time when *a*/2 leaf area has expanded and *b* is related to the maximum rate of leaf production. The maximum rate of leaf expansion (R_{max}, m^2 d^{-1}) was calculated from the first derivative of the logistic model at d_0 as $R_{max} = a/(4b)$. Leaf dry mass per area (LMA, g m^{-2}) was calculated as the ratio of dry mass and total leaf area. Assuming that LMA did not vary over time during the period of maximum expansion rate, we calculated plant growth rate (G, g dry mass d^{-1}) from R_{max} and LMA.

In all experiments, leaf traits were measured on a mature, fully-exposed but non-senescing leaf. The lamina was detached from the rosette, kept in deionised water at 4 °C for 24 h for water saturation, and then weighted (mg). After the determination of water-saturated mass, individual leaves were scanned for determination of leaf lamina area (LA, mm2) using ImageJ (https://imagej.nih.gov/ij/). Dry mass of the leaf lamina was obtained after drying for 72 h at 65 °C. Leaf dry matter content (LDMC, mg g^{-1}) and specific leaf area (SLA, mm^2 mg^{-1}) were calculated as the ratio of lamina dry and water-saturated mass, and the ratio of lamina area to lamina dry mass, respectively (Pérez-Harguindeguy et al., 2016).

Dry leaf blades were ground to determine N concentration (LNC, %), C concentration (LCC, %), and N and C isotopic ratio ($\delta^{15}N$ and $\delta^{13}C$, respectively) by mass spectrometry (EA2000, Eurovec, Isoprime, Elementar).

We calculated CSR scores (*i.e.* % along C, S, and R axes; see main text) based on three traits: LA, LDMC, and SLA, using the recent method developed by Pierce et al. (2017). The method is based on an algorithm which combines data for three leaf traits

(LA, SLA, and LDMC) that were shown to reliably position the species on the CSR scheme. We calculated CSR scores for each accession using average trait value per experiment using the calculation table provided in the Supplementary Information of Pierce et al. (2017).

In the 'PlastEdge' experiment, plant survival was measured directly after the temperature treatments. An individual was considered as alive if at least the center of its rosette was still green. We estimated pre-treatment mortality by analyzing pictures of the plate the day before treatment settlement. Individuals that did not germinate or died before the treatments were discarded from the analysis.

Metabolite quantitative measurement

Metabolite analyses were done with GC/MS for carbohydrates and plant hormones and LC/MS for glucosinolates, organic acids, and secondary metabolites (n = 124 per metabolite). For LC/MS analysis plant material was stored at -80° C prior to lyophilization, followed by homogenization with a ball mill (twice for 30 sec at 30 Hz). Extraction was done with 500 µl 80 % methanol, followed by a second extraction with 500 µl 20 % methanol containing 0.1 % formic acid. Both supernatants were combined and dried down in a vacuum concentrator. The resulting pellets were redissolved in 100 µl 20 % methanol containing 0.1 % formic acid. 5 µl were separated on a Waters Acquity UPLC system, equipped with a Waters Acquity HSST3 100 x 2.1mm, 1.8 µm column. Metabolite detection was done in ESI positive and negative mode with a Waters SynaptG2 mass spectrometer, scanned from m/z 50 to 2000 at a scan rate of 0.5 sec, and operated in MS and MS^E mode in parallel. Chromatography was performed at a flow rate of 200 µl per min with a 5 min gradient from 5 % water to 99 % methanol (both solvents containing 0.1 % formic acid) in ESI

negative mode and a 10 min gradient from 99 % water to 99 % methanol (both solvents containing 0.1 % formic acid) in ESI positive mode. Metabolite identification is based on accurate mass, fragmentation pattern, and standard material when available. For quantification, extract ion chromatograms were generated and integrated.

For carbohydrate analysis, plant material (n = 124) was stored at -80° C prior to homogenization with a ball mill (twice for 30 sec at 30 Hz). Extraction was done with 400 µl cold 80 % methanol containing 0.1 % formic acid and 3.9 µM 3-o-methylglucose as an internal standard followed by a second extraction with 400 µl cold 20 % methanol containing 0.1 % formic acid and 3.9 µM 3-o-methylglucose. 300 µl of both supernatants were combined, brought to dryness in a vacuum concentrator, and afterwards derivatized with 50 µL methoxamine (20 mg/ml in pyridine for 90 min at 30 °C), followed by a second derivatization step with 70 µl MSTFA for 30 min at 40 °C. From the resulting 120 µL, 60 µL were transferred into a new vial and 1 µl was injected onto a Shimadzu TQ 8040 GC/MS system operated at a splitting ratio of 1:100. Compound separation was achieved with a Restek Rxi-5SIL-MS glass capillary column (diameter of 0.25 mm, film thickness of 0.25 µm and a length of 30m). The carrier gas was helium at 1.13 mL/min column flow and a controlled linear velocity of 41.2 cm/sec. The oven program started at 60°C and was held for 3 min. Then the oven temperature increased with a rate of 10 K/min to a final temperature of 320°C which was held for 10 additional minutes. The mass spectrometer was operated in electron impact ionization (EI) mode. For compound detection, scan and selected ion monitoring (SIM) modes were used in parallel. The SIM chromatograms were

integrated and resulting peak areas were converted into absolute amounts by external calibration.

For plant hormone analysis, frozen plant material was grounded to fine powder in a ball mill and afterwards immediately extracted with 1.5 ml ethyl acetate, containing 0.1% formic acid and the internal standards 3-hydroxybenzoeic acid, dihydro-jasmonic acid, and 5IFA (40 ng, 50 ng, and 30 ng/ml respectively). Samples were incubated at 28° C for 60 min after a 10 min sonification step in an ultrasonic bath. After centrifugation at 18,500 g, 1.2 ml supernatant was transferred into a new tube. The ethyl acetate was removed to dryness in a gentle stream of nitrogen. Derivatization was done with a 1:1 mixture of 70 µl TMSDM (2.0 M in diethyl ether and methanol for 20 min at 25 °C. The reaction mixture was again evaporated under a mild nitrogen stream. Afterwards, samples were resuspended in 70 µL Hexane. Determination of analytes in 1 µl injected volume was performed by GC/MS (Shimadzu TQ8040), using splitless injection mode and a Restek Rxi-17SIL-MS column (30 m, 0.25 mm internal diameter, 0.25 µm film). The GC oven temperature was held at 70 °C for 5 min, then increased at a rate of 15 °C/min to 270 °C, then increased at a rate of 75 °C/min to 280 °C, and then held for additional 10 min at 280 °C. Helium was used as carrier gas with a flow rate of 1 ml/min. The mass spectrometer was operated in electron impact ionization (EI) and multiple reaction monitoring (MRM) mode. External calibration was used to convert MRM peak areas into absolute amounts.

Statistical models to predict trait values and plant categories

For all traits, pretreatments, calibration, and validation were carried out using python language (v3.6, https://www.python.org) with a Keras framework (v2.1.5,

https://keras.io/) and a TensorFlow backend (v1.6.0, https://www.tensorflow.org) for convolutional neural network (CNN) and scikit-learn (v0.24.2, https://scikit-learn.org/stable/) for partial least squares (PLS). Samples were divided into a calibration set (3/4) and a validation set (1/4) using Kennard-Stone algorithm (Kennard and Stone, 1969). For both PLSR and CNN, 12 filters (Table S2) were combined two by two. No spectral outlier was removed. Calibration was done minimizing the mean square error.

For PLSR, a three fold cross-validation step was applied on the calibration set to identify the best combination of pretreatments and number of components to retain. Calibration was then done on the entire calibration set using the identified pretreatments and number of components. An independent validation was finally done using the validation set.

For the CNN approach, a data augmentation procedure was applied on the calibration data set: for each original sample, five synthetic spectra were generated using a combination of random transformations of the original spectra and added to the initial calibration data set. Then all pretreatments were applied to this augmented dataset and the resulting spectra associated with the original ones. A convolutional neural network composed of three convolutional layers followed by two dense layers was fitted to the calibration data. Binary cross entropy was used as a loss function. In order to avoid overfitting, a batch normalization procedure/layer was applied between the first two convolutional layers and a dropout of 20% of features after the third layer. The model was calibrated using three fold cross validation. As for the PLSR approach, an independent validation was finally done using the validation set.

For the classification process, a data augmentation procedure was applied to generate up to 30 synthetic spectra for each original sample in order to rebalance classes. Binary cross entropy was used as a loss function.

Table S2: Details of base filters used for spectra pretreatment.

Code	Filter	Function/ Library	Arguments
Ga1	1D Gaussian filter	gaussian_filter1d /scipy	order=2, sigma=1
Ga2			order=0, sigma=2
Ga3			order=1, sigma=2
Ga4			order=1, sigma=1
Ha1	Haar transform	dwt/pywavelets	wavelet ='haar', mode = 'per', order=1
Ha2			wavelet ='haar', mode = 'per', order=2
MSC	Multiplicative scatter correction	polyfit /numpy	deg=1
Sg1	Savitzky-Golay filter	savgol_filter /scipy	window_legnth=17, polyorder=2, deriv=2, mode='interp'
Sg2			window_legnth=5, polyorder=2, deriv=0, mode='nearest'
Sg3			window_legnth=5, polyorder=2, deriv=0, mode='mirror'
Sg4			window_legnth=5, polyorder=2, deriv=0, mode='interp'
SNV	Standard Normal Variate	numpy	(x-mean(x))/std(x)

Pearson's coefficients of correlations (*r*) were calculated between observed trait values and predicted trait values. Regression lines were drawn from standard major axis (SMA) using the package *smatr*. All analyses were performed in R 3.2.3 (Team, 2014).

Table S3: Comparison of predictive power between PLSR and CNN.

	PLSR				CNN	
	**transformation**	**ncomp**	**RMSE**	r^2	**RMSE**	r^2
LNC (%)	ga1msc	6	0.80	0.83	0.52	0.93
δ13C	snvga1	4	0.87	0.65	0.61	0.83
Plant growth rate (mg d^{-1})	ha2ha2	4	0.00	0.51	0.00	0.57
R score (%)	mscha1	5	10.08	0.59	4.79	0.87
Fructose (µmol/g)	ga1	31	33.67	0.11	6.62	0.67
Maltose (µg/g)	ga1	3	53.86	0.02	55.53	0.05
Mannose (µmol/g)	ga2sg1	10	0.16	0.19	0.10	0.49
Ribose (µg/g)	sg1ga3	16	38.34	0.07	42.17	0.01
Arabinose (µg/g)	ga1snv	3	309.23	-0.01	51.42	0.01
JA (nmol/g)	snvga1	4	0.98	0.15	0.88	0.33
Glucoraphenin (Peakarea/mg)	ga2sg1	7	0.65	0.70	0.61	0.75
Hexyl glucosinolate	sg1ga3	14	54.04	-0.22	45.55	0.00
Butyl glucosinolate	ga4sg4	13	4.12	0.46	3.17	0.56
X3MTP (Peakarea/mg)	ga2ga1	3	41.90	-0.01	7.90	0.74
Dihydro caffeyol glucuronide	ga1snv	5	12.06	0.81	8.47	0.87

References

Estarague, A., Vasseur, F., Sartori, K., Bastias, C., and Cornet, D. (2021). Into the range: a latitudinal gradient or a center-margins differentiation of ecological strategies in Arabidopsis thaliana? *bioRxiv*. Available at: https://www.biorxiv.org/content/10.1101/2021.10.15.461205.abstract.

Granier, C., Aguirrezabal, L., Chenu, K., Cookson, S. J., Dauzat, M., Hamard, P., et al. (2006). PHENOPSIS, an automated platform for reproducible phenotyping of plant responses to soil water deficit in Arabidopsis thaliana permitted the identification of an accession with low sensitivity to soil water deficit. *New Phytol.* 169, 623–635.

Kennard, R. W., and Stone, L. A. (1969). Computer Aided Design of Experiments. *Technometrics* 11, 137–148.

Pérez-Harguindeguy, N., Diaz, S., Garnier, E., Lavorel, S., Poorter, H., Jaureguiberry, P., et al. (2016). Corrigendum to: New handbook for standardised measurement of plant functional traits worldwide. *Aust. J. Bot.* 64, 715.

Pierce, S., Negreiros, D., Cerabolini, B. E. L., Kattge, J., Díaz, S., Kleyer, M., et al. (2017). A global method for calculating plant CSR ecological strategies applied across biomes world-wide. *Funct. Ecol.* 31, 444–457.

Sartori, K. F. R., Vasseur, F., Violle, C., Baron, E., Gerard, M., Rowe, N., et al. (2018). Leaf economics guides slow-fast adaptation across the geographic range

of A. thaliana. *bioRxiv*, 487066. doi:10.1101/487066.

Sartori, K. F. R., Violle, C., Vile, D., Vasseur, F., de Villemereuil, P., Bresson, J., Gillespie, L., Fletcher, L.R., Sack, L. and Kazakou, E. (2022). Do leaf nitrogen resorption dynamics align with the slow-fast continuum? A test at the intraspecific level. *Funct. Ecol.* 00, 1–14

Team, R. C. (2014). R: A language and environment for statistical computing. R Foundation for Statistical Computing, Vienna, Austria.

Vasseur, F., Sartori, K., Baron, E., Fort, F., Kazakou, E., Segrestin, J., et al. (2018). Climate as a driver of adaptive variations in ecological strategies in Arabidopsis thaliana. *Ann. Bot.* doi:10.1093/aob/mcy165.
